# The Effects of Traffic Air Pollution in and around Schools on Executive Function and Academic Performance in Children: A Rapid Review

**DOI:** 10.3390/ijerph19020749

**Published:** 2022-01-10

**Authors:** Nicola Gartland, Halah E. Aljofi, Kimberly Dienes, Luke Aaron Munford, Anna L. Theakston, Martie van Tongeren

**Affiliations:** 1 School of Health Sciences, University of Manchester, Manchester M13 9PL, UK; halah.aljofi@postgrad.manchester.ac.uk (H.E.A.); luke.munford@manchester.ac.uk (L.A.M.); anna.theakston@manchester.ac.uk (A.L.T.); martie.j.van-tongeren@manchester.ac.uk (M.v.T.); 2 School of Psychology, Swansea University, Swansea SA2 8PP, UK; k.a.dienes@swansea.ac.uk

**Keywords:** traffic-related air pollution, cognitive function, working memory, school, children, academic achievement, review

## Abstract

This review summarises the extant literature investigating the relation between traffic-related air pollution levels in and around schools and executive functioning in primary-school-aged children. An electronic search was conducted using Web of Science, Scopus, and Education Literature Datasets databases (February 2020). Review articles were also searched, and forwards and backwards searches of identified studies were performed. Included papers were assessed for quality. We included 9 separate studies (published in 13 papers). Findings suggest that indoor and outdoor particulate matter with a diameter of 2.5 μm or less (PM_2.5_) negatively influences executive function and academic achievement and that indoor and outdoor nitrogen dioxide (NO_2_) adversely affects working memory. Evidence for the effects of particulate matter with a diameter of 10 μm or less (PM_10_) is limited but suggests potential wide-ranging negative effects on attention, reasoning, and academic test scores. Air pollution in and around schools influences executive function and appears to impede the developmental trajectory of working memory. Further research is required to establish the extent of these effects, reproducibility, consequences for future attainment, and place within the wider context of cognitive development.

## 1. Introduction

Air pollution not only affects respiratory and cardiovascular systems; it also has been shown to have significant effects on the central nervous system [1,2]. Magnetic resonance imaging (MRI) data demonstrate that air pollution is associated with damage to the prefrontal cortex and altered neurodevelopment in a variety of areas in children [3]. Neurological effects of air pollution provide a biologically plausible route to disruption of cognitive function, and research into the effects of air pollution on neuropsychological and cognitive outcomes throughout the life course is accumulating [4]. Here, we review the evidence investigating the relation between air pollution in and around schools and children’s cognitive and academic outcomes.

### 1.1. Traffic-Related Air Pollution

Traffic emissions (such as engine exhaust, brake, and tyre wear) are responsible for a large proportion of ambient air pollution; it is estimated that 46% of nitrogen oxides (NO_x_) emissions in EU countries come from transport. In terms of particulate matter (PM), road transport is reported to account for 13% of PM_10_ (PM with a diameter less than 10 μm) emissions and 15% of PM_2.5_ (PM with a diameter less than 2.5 μm) emissions [5]. PM is a mixture of solid and liquid particles suspended in the air that is categorized into three sizes: coarse particles (PM_10_), fine particles (PM_2.5_), and ultra-fine particles (UFP < 0.1 μm) [6]. The health implications from inhalation to these particle size fractions varies, with fine particles posing a greater risk to health than coarse particles [6]. In addition to the emission of PM, road transportation emits additional potentially harmful pollutants such as polycyclic aromatic hydrocarbons (PAHs) and NO_x_.

Children are more susceptible to the impact of air pollution than adults for a number of reasons. Children breathe more air per unit of body size because they have a higher breathing rate and are more physically active [7]. They also have increased exposure because they spend more time outdoors during peak traffic times, such as during school playtime. Young children play closer to the ground, where PM is more concentrated [8]. Children spend up to 8 h a day at school; therefore, schools represent an important and modifiable location for the study of the effects of pollution on children. Extensive research has looked at the effects of air pollution in and around schools on children’s health [9], but less is known about its impacts on their cognitive development and academic achievement.

There has been increased attention on the quality of the school environment due to the increased understanding of the effects of various aspects of this environment on health and achievement, including factors such as air quality, ventilation, lighting, and moisture [10,11,12]. Both indoor and outdoor levels of air pollution are pertinent and are related [12,13]. However, correspondence between these measures depends on multiple factors including type of building and windows, ventilation, level of outdoor pollution, and indoor air pollution sources [14].

### 1.2. Air Pollution and Health

Systemic inflammation and oxidative stress are possible biological mechanisms underlying the negative effects of air pollution on health and cognition. Both animal and human research demonstrates that airborne pollution inhaled through the nose can translocate to the olfactory bulb and migrate to the olfactory cortex, causing tissue damage and local inflammation [15,16]. Research assessing the effects of air pollution on children provides evidence that exposure to air pollution results in increased inflammation in the brain and the breakdown of the blood–brain barrier [17,18,19]. Air pollutants activate the body’s immune system, elevating cytokine expression and stimulating an inflammatory response. The resulting neuroinflammation carries an increased risk of neurodegeneration (cell loss), particularly if the inflammatory response occurs chronically; this is a likely mechanism by which executive function is affected [20]. The brain regions related to executive functions, including the pre-frontal cortex and the striatum [21,22], have shown inflammatory responses after exposure to traffic related air pollution (TRAP) [23].

Exposure to air pollution is also associated with increased levels of reactive oxygen species (ROS), either directly from PM or from particle-induced ROS formation. ROS play a crucial role in cellular processes, but an excess of ROS results in oxidative stress, which has been associated with neurodegenerative disorders [23,24]. Furthermore, there is evidence for altered brain activity in humans exposed to diesel exhaust, as measured by electroencephalogram [25].

### 1.3. Executive Function

Air pollution exerts a number of neurological effects, and the cognitive consequences of these effects for children is the topic of the current review. One method of assessing young children’s cognitive abilities is to measure executive function. Executive function is a collective term for a range of cognitive processes that manage and control thoughts, emotions, and actions aimed at achieving an objective or goal (e.g., working memory, impulse inhibition, cognitive flexibility, and planning). These functions are highly relevant to academic performance because they are fundamental for language development and literacy, as well as for the processing and organisation of new information. Essentially, they form the basis of a child’s ability to learn [26,27].

Working memory is the most studied of the executive functions. It is defined as ‘a limited capacity system allowing the temporary storage and manipulation of information necessary for such complex tasks as comprehension, learning and reasoning’ [28] (p. 418). There are multiple aspects to working memory, including (a) selective awareness, which constantly monitors information and identifies relevant and useful information for the task at hand, (b) sensitivity to distractions, which allows for the simultaneous performance of tasks, and (c) manipulation and transformation of information to orchestrate cognitive processes such as language comprehension, reasoning, and mathematical calculation [29,30]. Studies of working memory show that it develops rapidly during childhood and plateaus during adolescence [31,32], and there is evidence to support the suggestion that working memory around age 8 is related to academic performance in language and mathematics [33].

Attentional control covers a range of attentional processes which include the capacity to direct attention to specific stimuli and ignore irrelevant information, the ability to focus attention for an extended period, and the monitoring of errors and progress towards goals [34]. Two attention sub-systems have been proposed to develop during early childhood [35]. The first is the orienting system, which helps children to orient to stimuli in the external environment and to shift attention. The second, the anterior attention sub-system, selects and enhances processing by inhibiting and facilitating the orienting sub-system. These emerging systems contribute to a child’s ability to selectively attend to and focus on tasks.

Childhood is a time of significant development for executive function, and specific functions demonstrate different trajectories; attentional control shows rapid development in early childhood up to about 6 years of age [35], while cognitive flexibility (including working memory), goal setting, and information processing go through a significant period of development between 7 and 9 years of age [34].

It is recognised in the literature that academic performance is influenced by a wide range of variables including socioenvironmental, institutional, instructional, cognitive, and motivational factors. Executive function has been suggested to be central to academic achievement on the basis of its contribution to reading comprehension and literacy [27]. In a recent review and meta-analysis of the relation between executive functions and academic performance in primary education, Cortés Pascual and colleagues [30] report that executive functions are good predictors of academic achievement in typically developing children and that a range of executive function working memory was found to have the largest influence on academic performance. Longitudinal research has also demonstrated the predictive effects of executive functions (working memory, inhibition, and cognitive flexibility) at age 5 on academic achievement and classroom behaviour at age 8 [36]. Over a longer developmental period, Ahmed and colleagues [37] demonstrated that working memory at age 4 significantly predicts working memory and mathematics and reading achievement at age 15. The evidence suggests that while multiple executive functions are related to academic achievement, the specific function of working memory has a particularly long-lasting impact on academic success.

In summary, executive functions such as working memory and attention are essential for learning and achievement and develop significantly during childhood, particularly during a child’s time at primary school. The brain regions related to executive functions have shown inflammatory responses after TRAP exposure. The aim of this review was to summarise the existing evidence linking TRAP levels in and around schools with detrimental effects on executive functioning in primary aged children and consider the future academic impact of such a link. Specifically, we answer the following research questions:

1. Is executive function related to TRAP in and around schools?
2. Is academic achievement related to TRAP in and around schools?

## 2. Methods

An electronic search was conducted using Web of Science, Scopus, and Education Literature Datasets databases (February 2020), with the following search terms: air pollution, school, children, childhood, cognition, cognitive, executive function, education, achievement, attainment, neurodevelopment, memory. Review articles were also searched, and forwards and backwards searches of identified studies were performed.

Inclusion criteria included: (a) at least one continuous and quantitative measure of TRAP, (b) TRAP estimates at the school location, (c) at least one continuous and quantitative measure of cognitive or academic achievement. Exclusion criteria included: (a) only pollution exposure estimates outside of school setting, (b) only measurement of pollutants from non-traffic sources.

The papers included in the review were assessed for quality using a modified version of the Newcastle–Ottowa quality assessment scale for cohort studies [38]. Quality was rated on the representativeness of the cohort, estimates of the pollution exposure, measurement and modelling of appropriate control variables, measurement of outcome variable(s), and adequacy of follow-up measures of outcomes. Papers received a score out of nine, which reflected their relative overall quality. Papers rated as 6–9 were considered ‘high quality’; papers rated 5, ‘average quality’; and papers rated as 1–4, ‘low quality’.

## 3. Results

### 3.1. General Description of the Studies

We identified 9 studies for inclusion in the review, with a total of 13 associated papers published. Table 1 provides a descriptive overview of the papers included. The majority of the studies were based in Europe (five out of nine); one study entitled the BREATHE project was conducted in Spain (Barcelona) from which five papers were published, one in the UK (London), one in Austria (urban and rural regions), one in The Netherlands (Amsterdam), and one in Belgium (Flanders region). Three studies were conducted in the USA (one using national data, one in California, and one in Texas). One study was conducted in Chile (across three regions: Metropolitan, Valparaiso, and O’Higgins). Six of the studies focussed exclusively on primary aged children up to 11 years of age, with two of these studies including participants as young as 6. Two studies included some older students, up to 13 years and 16 years [39,40]. One final study measured overall school performance of ‘K–12′ schools, which covers ages 5–18 [41].

The papers report on a range of different traffic-related air pollutants (PM_2.5_, PM_10_, elemental carbon (EC), black carbon (BC), NO_2_, NO_x_, O_3_, CO, PAHs, and UFP). PM_2.5_ was the most studied pollutant, with exposure being measured or estimated in 8 out of 13 reviewed papers. NO_2_ was the second most studied pollutant, with exposure being measured or estimated in five of the reviewed papers. Exposure to PM_10_ was measured or estimated in four of the reviewed papers. There were two general ways in which pollutants were measured; six papers directly measured pollutants by sampling air at school sites indoors (two papers), outdoors (one), or both (three), while seven papers estimated pollutant exposure at school sites using geographically modelled pollution levels.

Five of the studies were cross-sectional in design, two studies used a cohort design (six papers; of which five utilised multiple measures of the outcome measure in analysis), and two were ecological studies where data were recorded at the school level rather than at the individual level.

There were two main categories of outcome measure. Five of the papers measured school attainment through standardised test scores (such as maths, reading, or reading comprehension scores), or grade point average (a summary score reflecting performance across all subjects throughout the school year, ranging from 0 to 4; GPA). The remaining nine papers investigated executive function through the completion of controlled psychological tests measuring working memory, attention, episodic memory, visual processing speed, reaction time, non-verbal reasoning, and coordination. The most commonly assessed executive functions were working memory and attention (reported in eight of these nine papers), and therefore, the variety of tests employed to assess these executive functions are outlined here:

Working memory was assessed by the computerised n-back task [34]. In this task, the subject is required to monitor a series of stimuli presented on screen and to respond whenever a stimulus is presented that matches the one presented in n trials previously (n = 1, 2, or 3). Higher n’s imply higher demands on working memory, and some studies referred to 2-back tests as assessing ‘working memory’ and 3-back tests as assessing ‘superior working memory’. Participants complete three blocks (1-, 2-, and 3-back) for each stimulus. Stimuli include colours, letters, numbers, and words. Various measures are taken for each trial, including accuracy measures (hits, correct rejections, false alarms, and misses) and hit reaction time (HRT). The most commonly used outcome for assessing working memory is the d prime (d’). d’ is computed as z (hit rate) − z (false alarm rate), with higher d’ indicating better signal detection and more accurate performance. Two papers also assessed working memory using the digit span task, which assesses forwards and backwards verbal recall of a sequence of digits presented auditorily and measures the number of digits that can be accurately recalled.

Attention was assessed in a few different forms in the reviewed papers. ‘Inattention’ is measured by the computerized Attentional Network Test (ANT) [52]. Reaction times (i.e., time between introduction of stimulus and reaction to stimulus) were measured and used to calculate the standard error of reaction time for correct responses (standard error of hit reaction time (HRT-SE)). HRT-SE is a measure of intra-individual variability reflecting response speed and consistency throughout the test. Higher HRT-SE scores reflect reduced executive and attentional resources. ‘Attention switching’ measures the ability to switch rapidly between responses. ‘Sustained attention’ is measured by the Continuous Performance Test and requires the respondent to quickly respond to relevant stimuli but ignore non-relevant stimuli. ‘Selective attention’ was measured by the Stroop Test.

There are many variables that may play a role in the expression of executive function in children. Therefore, the controlling of potential confounding variables is crucial for the valid interpretation of findings. Socioeconomic status (SES) is a particularly important variable to control for, as it is strongly related to cognitive and academic outcomes [53]. Twelve of the papers controlled for SES in some way (only one paper did not include any control variables in analysis [41]), and nine of the papers controlled for age, sex, mother’s education, and SES; five of these additionally controlled for pollution levels at residential locations (Table 1).

### 3.2. Quality Assessment

Quality scores ranged from two to seven, with an average of 4.6 (see Table 2). Seven papers were rated as low quality [39,40,41,45,47,48,51]. One paper was rated as average quality [43]. Five papers were rated as high quality [42,44,46,49,50]. The key points of distinction in the quality of the papers in the current review were (a) whether pollution was directly measured in or around schools or if it was estimated using databases of geographically mapped pollution levels, (b) whether executive function or academic outcomes were measured longitudinally or cross-sectionally, and (c) whether confounding variables such as SES, mothers’ education level, and residential pollution levels were controlled for. Papers that directly measured pollution levels, took repeated outcome measures, and controlled for relevant variables were rated as higher quality (Table 2).

### 3.3. Effects of Pollution on Executive Function

#### 3.3.1. Working Memory

Cross-sectional analysis of traffic pollution and working memory has reported mixed findings. Clark and colleagues [45] did not find a relation between estimated outdoor NO_2_ levels (using the King’s College London Emissions Toolkit) and working memory, as assessed by the Search and Memory Task in 9–10-year-olds. This was the only study that assessed working memory with this task. In contrast, another cross-sectional study which measured digit span length found that higher levels of estimated outdoor levels of NO_2_ were associated with reduced digit span length in 9–11-year-olds; NO_2_ levels did not affect any of the other neurobehavioural outcomes measured in the study [51]. The final cross-sectional study measuring working memory investigated the effects of same-day pollution levels (based on direct measurements of recent indoor PM_2.5_ and PM_10_) on a single assessment of working memory (digit span) in 8–11-year-olds and demonstrated no significant relation between the two [49]. Notably, these cross-sectional studies were carried out in North European countries, compared to the five cohort papers which were published from the BREATHE study based in Barcelona, Spain; therefore, climatic as well as pollution level differences may partly explain the differences in findings.

The findings from the papers originating from the BREATHE study modelled relative improvements in working memory in 7–10-year-olds over a set period of time; all five of these papers report significant negative relations between air pollution and working memory development. Alemany and colleagues [42] found that increased exposure to outdoor PAHs was associated with significantly less improvement in the 2-back numbers task over the course of a year but found no significant effect on the 2-back words test. Alvarez-Pedrerol and colleagues [43] also reported significantly smaller increases in working memory across one year (3-back numbers d’) with increased exposure to estimated outdoor BC and PM_2.5_ on the walk to school. Higher levels of indoor PM_2.5_ from traffic sources were specifically associated with less improvement in working memory (2-back numbers d’) and superior working memory (3-back numbers d’) across one year, where higher exposure was related to a 22% reduction in annual improvement in working memory and a 30% reduction in annual improvement in superior working memory [44]. The effects of outdoor traffic pollution in this paper were found to be less marked than the indoor levels. However, the most comprehensive analyses of indoor and outdoor levels or air pollution on multiple aspects of executive function were carried out by Sunyer and colleagues [50]. They found evidence that both indoor and outdoor levels of EC and NO_2_ had significant detrimental effects on the development of both working memory (2-back numbers and words d’) and superior working memory (3-back numbers and words d’) across a year. In addition, indoor exposure to UFP (but not outdoor UFP exposure) also significantly affected development of working memory. Demonstrating the overall effect of air pollution, children at high-pollution schools had a smaller growth in cognitive development than children from the paired low-pollution schools (7.4% (95% CI 5.6–8.8%) versus 11.5% (95% CI 8.9–12.5%) improvement in working memory over 1 year, *p* = 0.0024). Importantly, differences in baseline measures of executive function were not observed, but there were significant differences at the 1-year follow-up. For example, after 1 year, the difference in working memory for a change from the first to the fourth quartile of indoor EC was 6.2 points (95% CI 2.0–11.0, *p* = 0.004), which equates to 13.0% (95% CI 4.2–23.1%) of the total growth.

Only one paper from the Barcelona study modelled the development of working memory beyond one year. Forns and colleagues [46] looked at the effects of indoor and outdoor pollution on working memory (3-back numbers d’) development across 3.5 years. They report negative associations between all the measured pollutants, indoor and outdoor (EC, NO_2_, PM_2.5_ from traffic sources and UFP) and working memory across this period. When comparing inter-quartile range increases in exposure to pollutants, annual change in working memory was found to improve 10.2% less (95% CI, −17.2, −3.3) with high indoor NO_2_ exposure and 20.4% less (95% CI, −30.1, −10.7) for high outdoor NO_2_ levels. This is an important finding as it suggests that the negative effects of air pollution are maintained across longer time periods.

#### 3.3.2. Attention

Attention was measured in six of the reviewed publications and was operationalised in multiple forms, such as inattention, attention switching, selective attention, and sustained attention; this variation in measurement hampers comparability of findings. The findings relating to attention are mixed and appear to vary with particular air pollutants. In a cross-sectional analysis of the effects of PM_2.5_ and PM_10_, Saenen and colleagues [49] reported significant effects of recent indoor levels on selective attention in 8–11-year-olds; an inter-quartile range increment in PM_2.5_ was associated with 42.7 ms longer reaction times, while an inter-quartile range increment in PM_10_ was associated with 50.2 ms longer reaction times. However, they found no effect on sustained attention. In a separate cross-sectional study, NO_2_ was found not to have an effect on attention switching in 9–11-year-olds [51].

The papers originating from the BREATHE study modelled relative improvements in inattention in 7–10-year-olds over a one-year period. Increased exposure to outdoor PAHs was associated with significantly less improvement in inattention over a year [42]. Furthermore, higher levels of indoor PM_2.5_ from traffic sources were associated with increases in inattention across one year for, equivalent to 11% of the annual change for inattention [44]. However, Alvarez-Pedrerol and colleagues [43] did not find any significant effects of estimated BC or PM_2.5_ exposure on inattention over a year.

In Sunyer and colleagues’ [50] assessment of indoor and outdoor pollution, they found that for indoor pollution, only levels of EC had a significant effect on inattention such that higher levels of EC were associated with less improvement in inattentiveness across the year. Outdoor levels of EC and UFP had a significant effect on inattentiveness such that higher levels were associated with less reduction in inattentiveness. Neither indoor nor outdoor NO_2_ influenced the development of inattention in this sample.

#### 3.3.3. Additional Cognitive Measures

Additional cognitive measures which have been studied in reference to TRAP include visual information processing speed, episodic memory, non-verbal reasoning, simple reaction time, and hand–eye coordination. It is important to note, though, that only one of these cognitive outcomes was measured in more than one study (‘visual information processing speed’ or ‘perceptual coding and attention’—both measured by the digit symbol test [49,51]), and different air pollutants are measured in these studies; therefore, the opportunity for comparisons is limited. Furthermore, all analyses were carried out cross-sectionally.

Saenen and colleagues [49] reported that recent indoor PM_2.5_ and PM_10_ levels were associated with significantly increased total latencies in visual processing speed; IQR (inter-quartile range) increments in PM_2.5_ and PM_10_ levels were associated with total latency increases of 2.05 s and 1.9 s, respectively. However, van Kempen and colleagues [51] found no effect of estimated outdoor NO_2_ levels on digit symbol test performance.

Van Kempen and colleagues [51] found no effect of estimated NO_2_ levels on simple reaction times or hand–eye coordination. In a separate study of estimated NO_2_ levels, Clark and colleagues [45] reported no significant associations between NO_2_ and facets of episodic memory (recognition memory, information recall, and conceptual recall).

A comprehensive assessment of indoor air pollution at nine different schools in Austria was carried out by Hutter and colleagues [47]. This study furthered understanding by detailing the specific effects of pollutants within the PM_2.5_ and PM_10_ groups. Cognitive functioning was operationalised as non-verbal reasoning (assessed by the Standard Progressive Matrices Test [54]). A significant but small negative correlation with cognitive performance was demonstrated for phenanthrene in PM_2.5_ (r = −0.097), but no significant effects were found between phenanthrene or benzo(a)pyrene in PM_10_ and cognitive performance.

#### 3.3.4. Effects of Pollution on Test Scores/Academic Attainment

Five papers in the current review did not measure executive function or cognition but rather assessed the link between air pollution and academic achievement as measured by standardised test results or school performance. These papers could provide evidence for a ‘direct’ route for the effects of pollution on academic achievement.

Assessing the effect of estimated annual NO_2_ levels in London, Clark and colleagues [45] reported no significant relation between NO_2_ and children’s reading comprehension as measured by the Suffolk Reading Scale 2 [55]. Marcotte [48] estimated outdoor levels of PM_2.5_ and O_3_ on days when children completed standardised reading and maths tests and reported that after controlling for weather conditions, increased levels of PM_2.5_ on the test day were associated with lower reading but not maths scores, while O_3_ did not have an effect on test scores. They estimate that increasing PM_2.5_ from an average day to one with unhealthy air would be expected to decrease performance on reading assessments by 2%.

Miller and Vela [40] looked at grade-level test results using a standardised test in Chile. They found that annual estimates of PM_10_, but not PM_2.5_, significantly predicted test scores; a 10% decrease in PM_10_ was estimated to increase maths scores by 0.16% and reading scores by 0.14%. Annual levels of ozone (O_3_) were also found to have a negative relation with reading test scores but not maths test scores. This study provides evidence for short-term effects (one-week estimates) of pollutants as well as summative measures of pollution over longer periods (annual estimates). The effects of pollution in the week of exams were such that reading scores were significantly reduced by higher levels of PM_10_, PM_2.5_, and NO_x_ (but not CO or O_3_), while maths scores were significantly reduced by higher levels of PM_10_ and NO_x_ (but not PM_2.5_, CO, or O_3_).

The above studies all look at the effects of pollution on single test scores. Another way of operationalising academic achievement is to look at summary scores such as GPA or school-level performance indices. The advantage of such measures is that the outcome measure reflects a wider range of performance and thus is more indicative of overall academic success; however, the ecological design makes it difficult to interpret what findings mean on an individual level. In Texas, USA, Grineski and colleagues [39] reported a significant relation between a calculated diesel PM risk estimate and GPA. They used multi-level modelling to demonstrate that pollution exposure at the school level was a significant predictor of individual GPA, even when controlling for school level co-variates (e.g., percentage of students eligible for free meals, student–teacher ratio, percentage of teachers with a master’s degree) and individual-level control variables (e.g., sex, age, mother’s education, mother’s English proficiency); an inter-quartile range increase in on-road diesel PM risk was associated with a 0.16 reduction in GPA equivalent to 4%. Another study looked at whole school performance rather than individual pupil performance by looking at the school-level academic performance index (API) for 510 schools in California, USA [41]. Using estimated annual mean concentrations of PM_2.5_ over 3 years, they demonstrated that higher PM_2.5_ levels were associated with significantly lower API.

## 4. Discussion

This review provides an overview of the research that has been carried out to investigate the association between air pollution in and around schools and executive function or academic achievement in children. There is evidence to support the hypotheses that air pollution from traffic sources has a negative effect on both the executive function and academic achievement of primary-school-aged children. However, effects are not universal, and findings made in certain contexts and environments may not generalise. The literature is limited, but a few high-quality studies have been carried out that provide a valuable indication of the extent of this relation and highlight areas for targeted further research.

The relation between TRAP in and around schools and working memory becomes stronger when working memory is assessed over longer time periods. Therefore, TRAP appears to hamper the developmental trajectory of working memory. This is an important finding, as it holds the potential for continued divergence in working memory with age and could have significant knock-on effects in achievement. However, it is unclear from this research whether the effects of pollution act in a particular developmental window or if the differential in the trajectory is the result of the cumulative effects of continued exposure to high levels of air pollution. In terms of counteracting these effects, it is not yet known whether this divergence in working memory represents a lag in development which is eventually caught up or if it can be recovered through air pollution interventions. Further longitudinal research investigating the effects of interventions to reduce levels of air pollution are needed in order to delineate these effects. From the evidence available, PM demonstrates a negative relation with working memory, attention, and other cognitive outcomes, while NO_2_ may have a specific effect on working memory and may not affect other facets of executive function.

The lack of comparability across studies of the impact of TRAP on attention made it difficult to draw any firm conclusions. No studies reported an effect of NO_2_ or BC on attentional outcomes. There is evidence to support the suggestion that PAHs, EC, and UFP affect attentional outcomes and mixed findings for the effect of PM_2.5_. However, while four studies measured ‘inattention’, all of these were based on the BREATHE data from Barcelona, and none of the other studies measured this specific attentional variable. Indeed, all of the other studies measured different attentional variables to each other. Further research is needed to assess the immediate and long-term effects of TRAP on the various attentional processes.

The studies investigating the relation between air pollution and academic achievement support the suggestion that PM in particular has negative effects, while the evidence for the effects of NO_2_ and O_3_ is weaker. The problem with these studies is that they are few in number, and all are of lower quality: they use estimated pollution variables rather than taking objective measures within the school setting, most just look at one test score, none model longitudinal effects, and two of the studies were ecological in design, thus making it difficult to interpret what their findings mean on an individual level. Therefore, while these studies provide some insight into possible direct effects of air pollution on academic performance, it is also valuable to look at the ‘indirect’ effects of air pollution on executive function and use the high-quality studies assessing this relation to model the impact that these detriments may have on academic performance, given the strength of evidence linking working memory and academic performance [30,36,37].

Taken together, the evidence relating to PM_2.5_ suggests that it affects both executive function and academic achievement and that the effects become stronger over time. While the evidence relating to PM_10_ is limited, it suggests that PM_10_ has wide-ranging effects on attention, reasoning, and test scores. Interestingly, effects are not reported on working memory (while this is the main outcome affected by PM_2.5_), though this may be because it was not assessed in enough studies. Further research will be required in order to determine the full breadth of these effects. Only two studies rated high-quality measured levels of NO_2_; Sunyer and colleagues [50] and Forns and colleagues [46] both report negative effects of NO_2_ on long-term working memory but no impact on attention. Studies with lower quality ratings reported mixed findings regarding the relation between NO_2_ and cognitive outcomes. In summary, the limited evidence suggests that NO_2_ has a specific effect on working memory and may not affect other facets of executive function. However, this will need to be corroborated by further research.

Executive function is known to develop rapidly during childhood [33], and therefore longitudinal studies are best positioned to determine if the trajectories of executive function are affected by varying exposure to air pollution. Air pollution measurement also benefits from the implementation of longitudinal paradigms. In this review, air pollution was quantified variably as measurements taken on a single day or a recent (week-long) measure, measurements taken and modelled to provide a summative annual measure, and estimates of annual exposure based on land registry databases. Analyses of these different measures demonstrated a range of negative effects of air pollution; annual measures were shown to impact executive function, but short-term measurements were also shown to have relatively immediate effects on executive function [40,48,49]. Future research should aim to assess both these immediate and longer-term effects and how they interact with each other.

The evidence collected in the current review suggests that both indoor and outdoor levels of pollution impact children’s cognitive development. This is perhaps unsurprising, given the indoor and outdoor levels of TRAP are likely to be highly correlated. Interventions to reduce traffic and emissions near schools should take priority to improve the general health of school pupils as well as cognitive outcomes. This may not always be feasible, and indoor air filtration systems may provide a valuable opportunity for interventions to improve air quality [56] and thereby cognitive outcomes in this setting. However, other modifiable risk factors can also impact on children in school, such as indoor air quality, ventilation, and volatile organic compounds (VOCs) [57], and there are challenges to the accurate measurement of air pollution within schools [9]. Therefore, careful study design and the measurement of confounding variables will be essential for assessing the impact of changes to the environment within a school.

The long-term implications of detriments to executive function through exposure to air pollution have not yet been tested explicitly. Levels of working memory are significantly associated with achievement at school [30], and there is evidence for a predictive relationship between executive function and working memory, in particular in early childhood and performance through the rest of the educational system [36,37]. Therefore, there is potential for the effects of air pollution to have a significant and long-standing impact on academic achievement. However, longitudinal research measuring continued exposure to air pollution, executive function, and academic performance will be necessary to more accurately model the impact of air pollution on school success and success beyond education. Future research will need to determine whether these effects are sustained and if differentials in executive function on the basis of exposure continue to exist or if they converge or diverge further over time.

While we have focussed on the effects of pollution encountered within the school setting, it is important to recognise the wider range of exposure that children experience. Evidence suggests that the effects of pollution on brain development begin in utero [58,59] and have a significant long-term effect on cognitive development [60]. The effects of air pollution during infancy have significant effects on physical neurological development [3] and brain functioning. Residential air pollution and smoke exposure have also been shown to have a significant effect on children’s executive function [61,62]. Furthermore, TRAP can also be associated with noise pollution which can also impact cognitive function [63]. Therefore, pollution in schools is only one aspect of the exposure to pollution that any individual experiences. However, schools provide a focussed point of intervention where changes have the potential to influence a large number of children at once. These alternative sources of pollution exposure should be controlled for as thoroughly as possible in future research. Air pollution is ubiquitous in the modern world. Globally, annual PM levels were estimated to have increased by 8% between 2008 and 2013, and more than 80% of urban-dwelling people are exposed to air quality levels that exceed World Health Organisation limits (where air pollution is monitored) [64]. Therefore, while the impact of these effects on an individual child may appear small, the impact at the population level is much greater. The implications of these effects are far-reaching and are relevant to the global burden of air pollution. The effects of air pollution on the cognitive development of children represents only one small aspect of the impact of air pollution on our lives and environment, but it should be taken into consideration when issues about air pollution are examined.

## 5. Conclusions

Evidence supports the hypotheses that air pollution from traffic sources has a negative effect on both the executive function and academic achievement of primary-school-aged children. The population level implications for these findings are significant. However, effects are not universal, and findings made in certain contexts and environments may not generalise. The small number of studies identified, and the quality of the studies included, highlight the need for further research in this area. It will be particularly important to measure these longitudinal relationships in different settings to determine the variability of effects, as well as identifying key settings for targeted intervention.

## Figures and Tables

**Table 1:** Characteristics of reviewed papers.

Author and Country	Design	*n*	Age Range	Air Pollutants Investigated	Pollution Estimated/Measured	Outcome Measures	Control Variables	Results
1. Alemany et al., (2018) Barcelona, Spain [42]	Cohort	2897	7–11	Schoolyard pollution: Polycyclic aromatic hydrocarbons (PAHs) Elemental carbon (EC) Nitrogen dioxide (NO_2_)	Measured Summary measure	Behavioural problems Inattentiveness Working memory	age sex maternal education level residential neighbourhood SES	IQR increases in PAHs: Inattentiveness (β = 4.44; 95% CI: 0.48, 8.40) 2-back numbers d’ values (WM) (β = −0.08; 95% CI: −0.14, −0.02) 2-back words d’ values (WM) (β = −0.02; 95% CI: −0.07, 0.04)
2. Alvarez-Pedrerol et al., (2017) Barcelona, Spain [43]	Cohort	1234	7–10	Pollutants from walking commute to school: Average particulate matter ≤2.5 µm (PM_2.5_) Black carbon (BC) Nitrogen dioxide (NO_2_)	Estimated Summary measure	Inattentiveness Working memory	age sex maternal education level residential neighbourhood SES commuting time school and home air pollution	IQR increase in PM_2.5_: WM (β = −9.0, 95% CI (−15.5, −2.6), *p* < 0.01) IQR increase in BC: WM (β = −7.8, 95% CI (−13.4, −2.3), *p* < 0.01) No significant associations for inattentiveness.
3. Basagana et al., (2016) Barcelona, Spain [44]	Cohort	2618	7–10	Indoor and outdoor PM_2.5_ pollution at schools: Sulfate Nitrate Chloride Ammonium Organic carbon (OC) Elemental carbon (EC)	Measured Summary measure	Inattentiveness Working memory Superior working memory	age sex maternal education level residential neighbourhood SES air pollution exposure at home	IQR increase in indoor traffic source: WM (β = –5.6; 95% CI: –10.7, –0.5) SWM (β = –5.1; 95% CI: –9.2, –1.1) Inattentiveness (β = 3.6; 95% CI: 0.0, 7.1)
4. Clark et al., (2012) London, UK [45]	Cross-sectional	719	9–10	Outdoor pollution levels linked to school postcodes: NO_2_	Estimated Summary measure	Reading comprehension Episodic memory Working memory	age sex maternal education level parental employment status crowding in the home home ownership long-standing illness main language spoken at home parental support for schoolwork classroom window glazing	NO_2_ levels not significantly associated with reading comprehension, recognition memory, information recall, conceptual recall, or working memory (per 1-point increase in nitrogen dioxide (μg/m^3^)).
5. Forns et al., (2017) Barcelona, Spain [46]	Cohort	1439	11.4 (SD0.6) at last follow-up (3.5 years post-baseline)	Indoor and outdoor pollution at schools: Elemental carbon (EC) Nitrogen dioxide (NO_2_) Particulate matter (PM_2.5_) from traffic sources Ultra-fine particles (UFP)	Measured Summary measure	Working memory	age sex grade maternal education level Urban Vulnerability Index at home address air pollution exposure at home address (NO_2_)	IQR increase in NO_2_: WM (indoor β = −2.11; 95% CI: −3.54, −0.68; outdoor β = −4.22; 95% CI: −6.22, −2.22) IQR increase in EC: WM (indoor β = −2.92; 95% CI: −4.53, −1.31; outdoor β = −2.13; 95% CI: −3.26, −0.99) IQR increase in PM_2.5_: WM (indoor β = −3.38; 95% CI: −5.81, −0.95; outdoor β = −2.30; 95% CI: −3.65, −0.96) IQR increase in UFP: WM (indoor β = −4.12; 95% CI: −6.51, −1.73; outdoor β = −3.75; 95% CI: −5.68, −1.83)
6. Gaffron and Niemeier (2015) California, USA [41]	Ecological	553 schools with 250,433 students	NA	Outdoor air pollution linked to school location: PM_2.5_ Diesel particulate matter Traffic density	Estimated Summary measure	Test scores: School-level academic performance index (API)	No control variables.	PM_2.5_ levels correlated with API (r = −0.21, R2 = 0.04, *p* < 0.001)
7. Grineski et al., (2016) Texas, USA [39]	Ecological	1888	8–13	Outdoor respiratory and diesel particulate matter HAP risk estimates: Total diesel particulate matter (PM) On-road diesel PM Non-road diesel PM	Estimated Summary measure Risk estimate	Grade point average (GPA)	School-level control variables: total enrolment % free/reduced price meals student/teacher ratio % special education % teachers with MA degree Individual-level control variables: sex age free/reduced price meals teen mother mother’s education mother is Hispanic mother is Black mother’s English proficiency	IQR increase in total diesel PM risk: GPA (β = −0.22; 95% CI: −0.37, −0.07) IQR increase in on-road diesel PM risk: GPA (β = −0.16; 95% CI: −0.29, −0.04) IQR increase in non-road diesel PM risk: GPA (β = −0.11; 95% CI: −0.26, −0.05)
8. Hutter et al. (2013) Austria (rural and urban regions) [47]	Cross-sectional	436	6–8	Indoor pollution at schools: Particulate matter (PM_10_ and PM_2.5_) Carbon dioxide (CO_2_) Chemical parameters (252 different ones)	Measured	Non-verbal reasoning	Social status (parental education and occupation) gender region (urban/rural; population density)	TCEP (PM_10_) correlated with cognitive performance (r = −0.147, *p* = 0.003) TCEP (PM_2.5_) correlated with cognitive performance (r = −0.149, *p* = 0.002) No significant correlations between PM_10_ phenanthrene, benzo(a)pyrene, or TDCPP with cognitive performance. Phenanthrene concentrations (PM_2.5_) correlated with cognitive performance (r = −0.097, *p* = 0.047) CO_2_ correlated with cognitive performance (r = −0.102, *p* = 0.034).
9. Marcotte (2017) National data, USA [48]	Cross-sectional	1450	6.75 Mean in months 81.01 SD 11.57	Outdoor pollution levels linked to school locations: Ozone (O_3_) Particulate matter (PM_2.5_)	Estimated Multiple measures	Test scores: Maths score Reading score	family composition poverty status child gender race and ethnicity % students in grade eligible for free/reduced price meals high temperature precipitation common year/season fixed effects	PM_2.5_ significantly predicts reading score (β = −0.02, SE = 0.01, *p* < 0.05). No significant effect on maths score. No significant effects of O_3_ on test scores.
10. Miller and Vela (2013) Chile (Metropolitan, Valparaiso, O’Higgins) [40]	Cohort	3880 schools	10–16	Outdoor daily pollution levels linked to school locations between 1997 and 2012: Particulate matter (PM_10_ and PM_2.5_) Carbon monoxide (CO) Nitrogen oxide (NOx) Ozone (O_3_)	Estimated Daily measures of PM_10_, annual averages for other pollutants	Test scores: Maths score Reading score	Total children per class school SES public, private, or charter type	PM10 levels predict test scores (reading: β = −0.07, SE = 0.02, *p* < 0.01; maths: β = −0.08, SE = 0.02, *p* < 0.01). No significant effect of PM_2.5_. Effects in the week of exams: Significant effects on reading for PM_10_ (β = −0.14, SE = 0.01, *p* < 0.01), PM_2.5_ (β = −0.24, SE = 0.04, *p* < 0.01), and NOx (β = −0.18, SE = 0.04, *p* < 0.01). Non-significant effects of CO and O_3_. Significant effects on maths for PM10 (β = −0.12, SE = 0.01, *p* < 0.01) and NOx (β = −0.16, SE = 0.04, *p* < 0.01). Non-significant effects of PM_2.5_, CO and O_3_.
11. Saenen et al., (2016) Flanders, Belgium [49]	Cohort (analysed cross-sectionally)	310	8–11	Indoor classroom PM_2.5_ and PM_10_	Measured Multiple measures	Selective attention Sustained attention Short-term memory Visual information processing speed	sex age (linear and quadratic) education of the mother occupation of the parents passive smoking out-of-school physical activity traffic noise day/night hours of computer screen time per week day of the week relatedness of the examination periods chronic residential pollution exposure	Selective attention: IQR increase in PM_2.5_: 42.7 ms (95% CI: −0.40 to 85.8, *p* = 0.05) IQR increase in PM10: 50.2 ms (95% CI: 8.55 to 91.8, *p* = 0.02). Visual information processing speed: IQR increase in PM_2.5_ (2.05 s; 95% CI: 0.43, 3.66; *p* = 0.01). The IQR increase in PM10 was 1.9 s (*p* = 0.02). No significant associations between classroom PM and sustained attention or short-term memory.
12. Sunyer et al., (2015) Barcelona, Spain [50]	Cohort	2715	7–10	Indoor and outdoor pollution at schools: Elemental carbon (EC) Ultra-fine particles (UFP; 10–700 nm) Nitrogen dioxide (NO_2_)	Measured Summary measure	Inattentiveness Working memory Superior working memory	age sex maternal education residential neighbourhood SES air pollution exposure at home	INDOOR IQR increase in EC: WM (β = −6.2, (95% CI −11, −2), *p* < 0.05) SWM (β = −5.8, ( 95% CI −9.2, −2.4), *p* < 0.05) Inattentiveness (β = 3.9, (95% CI 0.79, 6.8), *p* < 0.05) IQR increase in NO_2_: WM (β = −5.6, (95% CI −11, −0.44), *p* < 0.05) SWM (β = −5.1, (95% CI −9.2, −0.91), *p* < 0.05) inattentiveness (β = 2.6, (95% CI −1.0, 6.3), NS) IQR increase in UFP: WM (β = −7.9, (95% CI −15, −1.3), *p* < 0.05) SWM (β = −6.0, (95% CI −11, −0.75), *p* < 0.05) Inattentiveness (β = 4.6, (95% CI −0.13, 9.2), NS) OUTDOOR IQR increase in EC: WM (β = −4.1, (95% CI −8.0, −0.2), *p* < 0.05) SWM (β = −4.4, (95% CI −7.6, −1.3), *p* < 0.05) Inattentiveness (β = 3.8, (95% CI 1.0, 6.6), *p* < 0.05) IQR increase in NO_2_: WM (β = −6.6, (95% CI −12, −1.2), *p* < 0.05) SWM (β = −6.7, (95% CI −11, −2.3), *p* < 0.05) Inattentiveness (β = 3.8, (95% CI −0.10, 7.6), NS) IQR increase in UFP: WM (β = −4.9, (95% CI −10, 0.22), NS) SWM (β = −5.0, (95% CI −9.1, −0.96), *p* < 0.05) Inattentiveness (β = 3.9, (95% CI 0.31, 7.6), *p* < 0.05)
13. van Kempen et al. (2012) Amsterdam, The Netherlands [51]	Cross-sectional	553	9–11	Outdoor air pollution linked to school: Nitrogen dioxide (NO_2_) Particulate matter (PM_10_)	Estimated Summary measure	Reaction time Attention switching Coordination Perceptual coding and attention Working memory	age sex crowding home ownership employment and mother’s education longstanding illness (y/n) parental support main language spoken at home is Dutch (y/n) type of window glazing at school	NO_2_ at school associated with WM (β = −0.16, 95% CI: −0.28 −0.04) No significant effects on other cognitive outcomes. Insufficient variability in levels of PM_10_ to test.

IQR—inter-quartile range; PM—particulate matter; SES—socioeconomic status; SWM—superior working memory; WM—working memory.

**Table 2:** Quality assessment of reviewed papers.

	Selection				Comparability	Outcome			
	Representativeness of the Sample	Measurement of Exposure	Modelling of Variation in Exposure	Measurement of Outcome at Start and End of Study Period	Controlling for Confounding Variables	Assessment of Outcome	Appropriate Length of Follow-Up	Adequacy of Follow-Up Sample	Quality Score (Max 9)
Alemany et al. [42]	*	*		*	**	*			6
Alvarez-Pedrerol et al. [43]	*			*	**	*			5
Basagana et al. [44]	*	*		*	**	*		*	7
Clark et al. [45]	*			NA	*	*	NA	NA	3
Forns et al. [46]	*	*		*	**	*	*		7
Gaffron and Niemeier [41]	*			NA		*	NA	NA	2
Grineski et al. [39]	*			NA	*		NA	NA	2
Hutter et al. [47]	*	*		NA	*	*			4
Marcotte [48]			*	NA	*	*	NA	NA	3
Miller and Vela [40]	*		*	NA	*	*	NA	NA	4
Saenen et al. [49]		*	*	*	**	*		*	7
Sunyer et al. [50]	*	*		*	**	*		*	7
van Kempen et al. [51]	*			NA	*	*	NA	NA	3

* indicates quality standard was met for these criteria; within ‘controlling for confounding variables’, ** could be achieved where two standards were met (controlling for residential pollution and controlling for additional factors).

## Data Availability

No new data were created or analyzed in this study. Data sharing is not applicable to this article.

## Abbreviations

ANT	Attentional Network Test
API	Academic Performance Index
BC	black carbon
CI	confidence interval
CO	carbon monoxide
EC	elemental carbon
GPA	grade point average
HRT	hit reaction time
HRT-SE	standard error of hit reaction time
IQR	inter-quartile range
NOx	nitrogen oxides
NO_2_	nitrogen dioxide
O_3_	ozone
PAH	polycyclic aromatic hydrocarbon
PM	particulate matter
ROS	reactive oxygen species
SES	socioeconomic status
TRAP	traffic-related air pollution
UFP	ultra-fine particle
VOC	volatile organic compound
